# Sensitive detection of low-abundance in-frame deletions in EGFR exon 19 using novel wild-type blockers in real-time PCR

**DOI:** 10.1038/s41598-019-44792-1

**Published:** 2019-06-04

**Authors:** Xiao-Dong Ren, Ding-Yuan Liu, Hai-Qin Guo, Liu Wang, Na Zhao, Ning Su, Kun Wei, Sai Ren, Xue-Mei Qu, Xiao-Tian Dai, Qing Huang

**Affiliations:** 1 Department of Laboratory Medicine, Southwest Hospital, Third Military Medical University (Army Medical University), Chongqing, 400038 P.R. China; 2 Department of Laboratory Medicine, Institute of Surgery Research, Daping Hospital, Third Military Medical University (Army Medical University), Chongqing, 400042 P.R. China; 3 Department of Pulmonology, Southwest Hospital, Third Military Medical University (Army Medical University), Chongqing, 400038 P.R. China

**Keywords:** Targeted therapies, Molecular biology

## Abstract

Epidermal growth factor receptor (EGFR) mutations are associated with response of tyrosine kinase inhibitors (TKIs) for patients with advanced non-small cell lung cancer (NSCLC). However, the existing methods for detection of samples having rare mutations(i.e. ~0.01%) have limits in terms of specificity, time consumption or cost. In the current study, novel wild-type blocking (WTB) oligonucleotides modified with phosphorothioate or inverted dT at the 5′-termini were designed to precisely detect 11 common deletion mutations in exon 19 of EGFR gene (E19del) using a WTB-PCR assay. And internal competitive *leptin* amplifications were further applied to enhance the specificity of the WTB-PCR system. Our results showed that WTB-PCR could completely block amplification of wild-type EGFR when 200 ng of DNA was used as template. Furthermore, the current WTB-PCR assay facilitated the detection of E19del mutations with a selectivity of 0.01% and sensitivity as low as a single copy. And, the results showed that the current WTB-PCR system exceeded detection limits afforded by the ARMS-PCR assay. In conclusion, the current WTB-PCR strategy represents a simple and cost-effective method to precisely detect various low-abundance deletion mutations.

## Introduction

Lung cancer is the leading cause of cancer mortality worldwide, accounting for one third of all cancer-related deaths^1^. Among the different lung cancer diseases, non-small cell lung cancer (NSCLC) is the most common, accounting for approximately 80–85% of all lung cancer cases^2^. Epidermal growth factor receptor (EGFR) is expressed in 50% of NSCLCs, and therefore serves as a molecular target in therapeutics for NSCLC patients^1^. Among the various small molecule inhibitors that specifically target the tyrosine kinase activity of EGFR (i.e., EGFR-tyrosine kinase inhibitors (EGFR-TKIs)), gefitinib and erlotinib are the most commonly used molecular-targeted therapeutics for NSCLC patients^1^. However, preliminary clinical data have revealed that only approximately 10% of unselected NSCLC patients responded to gefitinib or erlotinib^1^. Recently, various clinical studies have reported that the status of *EGFR* gene mutations at exons 18–2 l in the tyrosine kinase coding domain are correlated with the therapeutic response of both gefitinib or erlotinib for NSCLC patients^1,2^. *EGFR* can be divided into drug-sensitive (e.g., in-frame deletions of exon 19 and L858R substitution in exon 21) and -resistant (e.g., T790M in exon 20) mutations depending on the patient’s response to EGFR-TKI therapeutics^1^. The most prevalent EGFR kinase domain mutations are the in-frame deletions of exon 19 (E19del); these mutations account for approximately 45% of *EGFR* mutations in NSCLC patients. Another recurrent mutation is the L858R in exon 21, which account for approximately 40–45% of *EGFR* mutations. In unselected NSCLC samples, although *EGFR* mutations are present in ~10% of cases in North America and Western Europe, approximately 30–50% of these cases are of East Asian descent^1,3,4^. Therefore, it is important that *EGFR* mutations are detected while screening for drug-sensitive or -resistant NSCLC patients; this is particularly important for Chinese individuals who will undertake targeted therapeutics.

However, due to the intra-tumor heterogeneity, there are a small proportion of mutant cancer cells in clinically available tissue samples including formalin-fixed paraffin-embedded (FFPE) tissue sections; this phenomenon result in that the extracted DNA from FFPE contain excessive wild-type genomic DNA (WT-gDNA). Indeed, recent studies indicated that highly selective mutation assays can distinguish patients who had poor responses to anti-EGFR antibodies therapy in colorectal carcinomas^5,6^. Therefore, the development of high sensitive and selective methods to detect low-abundance *EGFR* mutations are urgently required.

Selectivity refers to the capacity to detect mutant (MT) gene among an excess of wild-type (WT) gene. The calculation method of selectivity is the ratio of copy number between MT-gene and the total gene including both WT- and MT-gene^7–10^. Currently, there are various methods available to analyze *EGFR* mutations; these methods include pyrosequencing, Sanger sequencing, amplification refractory mutation system (ARMS-PCR), allele-specific hydrolysis or dual hybridization probes, PCR restriction fragment length polymorphism (PCR-RFLP), high-resolution melting analysis (HRMA), next generation sequencing (NGS), wild-type blocking PCR (WTB-PCR), and droplet digital PCR (dPCR)^11–16^. However, most of these methods, apart from more recently developed methods including WTB-PCR and dPCR, exhibit limitations in the detection of *EGFR* mutations^11–16^. Compared with other available methods, low-abundance MT-allele analysis methods such as competitive-allele-specific TaqMan PCR (CAST-PCR), co-amplification at lower denaturation temperature PCR (COLD-PCR), LigAmp assay, BEAMing, IntPlex and dPCR, WTB-PCR is one of the most selective and sensitive methods^7,17–24^. In WTB-PCR, the wild-type blockers (WTBs) specifically hybridize to WT-gene, thereby blocking amplification of these gene and permitting the selective amplification of the MT-gene^7,17,25–27^. In traditional WTB-PCR, two types of WTBs have been employed. In one of these methods, one of the WTB oligonucleotides overlaps with one of the forward and reverse primers^25^. In the second WTB method, the WTB oligonucleotide is located between the forward and reverse primers^17,26,28^. In relation to the latter WTB-PCR strategy, the WTBs were always prepared as expensive peptide nucleic acids (PNA) capable of resisting the 5′ to 3′ exonuclease activity normally associated with *Taq* DNA polymerase. However, if other types of oligonucleotides are required, such as DNA, locked nucleic acids (LNA), or LNA/DNA chimera oligonucleotides, the DNA polymerase must be deficient in both strand-displacement and 5′ to 3′ exonuclease activities^26^.

In the present study, to avoid the 5′ to 3′ exonuclease activity of *Taq* DNA polymerase, LNA/DNA chimeras with modified functional groups (i.e., phosphorothioate modifications or inverted dT) at one or more of the 5′-terminal bases were used as WTB oligonucleotides to selectively eliminate the amplification of E19del WT-gene. Compared with PNA, LNA/DNA chimeras have been shown to be more cost-effective WTB oligonucleotides. Similarly, based on the 5′-modification of the WTB oligonucleotides used in this study, mutant-gene specific TaqMan hydrolysis probes (MST) could be used in WTB-PCR to clearly identify MT-gene. Moreover, to satisfy the thermodynamic driving force of DNA polymerase, an internal competitive amplified fragment (i.e., human *leptin* gene) was introduced in the reaction mixture to further increase the specificity of the current WTB-PCR system. The results showed that the current WTB-PCR system is a reliable and simple method that can be used to quantitatively detect E19del mutations with high selectivity (i.e., 0.01%) and sensitivity (i.e., single copy). Compared with commercially available ARMS-PCR, an additional 10% (6/62) of FFPE samples from NSCLC patients were shown to harbor E19del mutations using the WTB-PCR system. Because the proportion of mutant gene of the afore-mentioned 6 positive samples exceeded the detection limits of the ARMS-PCR system.

## Materials and Methods

### Extraction of sample genomic DNA

The FFPE samples used in this study were derived from 62 patients who had been diagnosed with advanced NSCLC; all of the patients had signed informed consent (Supplementary Table 1). The HCC827 cell line (ATCC, Manassas, VA, USA) used in this study harbored one of *EGFR* E19del mutations (i.e. E746-A750del)^29^. The genomic DNA (gDNA) from FFPE tissue sections, HCC827 cell lines and the whole blood of healthy volunteers was extracted with the QIAamp DNA FFPE Tissue Kit (Qiagen, Hilden, Germany) or the QIAamp DNA Blood Mini Kit (Qiagen) following the manufacturer’s introductions. The FFPE tissue blocks from Chinese NSCLC patients were obtained from the Southwest Hospital (Chongqing, China). The ethics committee of Southwest Hospital approved this study including any relevant details, and written informed consent was obtained from the patients or their family members prior to sample collection. All experiments were performed in accordance with relevant guidelines and regulations.

### Quantification of extracted gDNA

The concentration of the isolated gDNA was quantitatively determined using the *leptin* quantitative PCR (qPCR) system as previously described^7^. The 20 μL reaction mixture contained 1x Platinum® Quantitative PCR SuperMix-UDG (Invitrogen, Waltham, MA, USA), 200 nM of each of the Lf- and Lr-primers and TaqMan hydrolysis probes (L-probe; Table 1). Serial concentrations of the human gDNA (Cat No. G304A; Promega, Madison, WI, USA) isolates were used to prepare a standard curve for the *leptin* gene. Reactions were performed on a CFX96 Real-Time PCR Detection System (Bio-Rad, Hercules, CA, USA) under the following cycling conditions: incubation at 50 °C for 2 min, denaturation at 95 °C for 30 sec, and 50 cycles of 95 °C for 15 sec and 60 °C for 30 sec. The quantification cycle (*C*_q_) values were determined automatically using CFX Manager™ Software v3.1 (Bio-Rad)^7^.

**Table 1:** Oligonucleotides used in the current study.

Targets	ID	Description	5′-3′ Sequences
*EGFR*	SW-1630	Ef-primer	gttaaaattcccgtcgctatcaa
	SW-1631	Er-primer	agcagaaactcacatcgaggatt
	SW-1632	E-probe	(6-FAM) cgaaagccaacaagg (MGB)
	SW-1633	WTB-1	gaattaagagaagca (MGB)
	SW-1718	WTB-2	*gAATTAAGAGAAGCA (C3-spacer)
	SW-1713	WTB-3	*g*g*aATTAAGAGAAGca (C3-spacer)
	SW-1712	WTB-4	*g*g*aATTAAgAGAAGca (C3-spacer)
	SW-1717	WTB-5	^#^tAATTAAGAGAAGCA (C3-spacer)
*leptin*	SW-329	Lf-primer	cagtctcctccaaacagaaagtca
	SW-330	Lr-primer	gtccatcttggataaggtcagga
	SW-1434	L-probe	(VIC) cggtttggacttcatt (MGB)

*Phosphorothioate modification; ^#^Inverted dT; MGB: minor groove binder; C3-Spacer: Spacers modifications at the 3′-terminal base; Ef-primer, Er-primer and E-probe represent forward primers, reverse primers and probe targeting *EGFR* gene respectively; Lf-primer, Lr-primer and L-probe represent forward primers, reverse primers and probe targeting *Leptin* gene respectively. The upper-case letters indicate LNAs. WTB-1 to -5 represent 5 types of WTBs targeting *EGFR* E19del in-frame deletions. The amplicon size of targeted *EGFR* and *leptin* were 85 and 80 bp, respectively.

### Preparation of quality control (QC) plasmids

**Figure 1:**
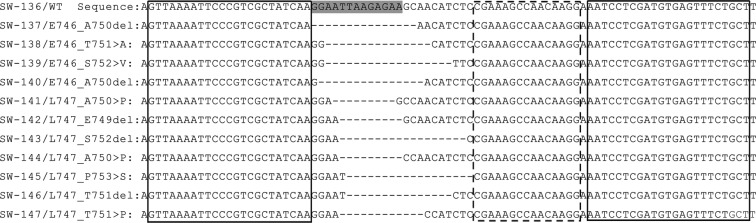
Sequence alignments of WT- and MT-alleles for the in-frame deletion of *EGFR* E19del. The solid-line boxes indicate the positions of the Ef- and Er-primer, The dotted-line boxes indicate the positions of the LST oligonucleotides. The gray-highlighted letters indicate the positions of the WTB oligonucleotides. The short dotted-lines indicate the in-frame deleted sequences for each E19del in the corresponding MT-QC plasmids. The WT-QC plasmid was entitled “SW-136” while the MT-QC plasmids were entitled SW-137 to -147; the latter plasmids represented the 11 types of E19del hot mutations, respectively. The sequencing chromatographs of the afore-mentioned QC plasmids are presented in Supplementary Fig. 2.

Various E19del WT- and MT-gene fragments (Fig. 1) of similar size were artificially synthesized by Sangon Biotech Co. Ltd. (Shanghai, China) and then serially cloned into a plasmid to prepare WT- and MT-QC plasmids. The sequences of all QC-plasmids were confirmed by DNA Sanger sequencing. In total, one WT-QC plasmid and 11 MT-QC plasmids harboring the hot mutations of *EGFR* E19del were generated^1,30^.

### Screening various types of WTBs and optimization of the reaction system

The single factor variable method was used to screen and optimize various WTBs (WTB-1 to 5 in Table 1) using the current WTB-PCR system. In this study, 5 different types WTBs were used in WTB-PCR which including traditional WTBs that could not resist the 5′ to 3′ exonuclease activity of Taq DNA polymerase (i.e., WTB-1 in Table 1). WTBs having one or more phosphorothioate modifications (i.e., WTB-2 to -4 in Table 1) or one inverted dT (i.e., WTB-5 in Table 1) at the 5′-terminal bases to avoid its hydrolysis under the 5′ to 3′ exonuclease activity of Taq DNA polymerase. Serial concentrations of various WTBs were added into reaction mixtures containing specific concentrations of WT- or MT-templates that were prepared using WT- and MT-gDNA, or WT- and MT-QC plasmids. The reaction mixtures contained 1x Platinum® Quantitative PCR SuperMix-UDG (Invitrogen) with 900 nM each of the Ef- and Er-primer targeting *EGFR*, 400 nM E-probes targeting *EGFR*, serial concentrations (0, 200, 500, and 800 nM) of various WTBs targeting *EGFR*, and specific concentrations of WT- or MT-templates. The reactions were performed on a CFX96 Real-Time PCR Detection System (Bio-Rad) under the following cycling conditions: incubation at 50 °C for 2 min, denaturation at 95 °C for 30 sec, and 50 or 60 cycles of 95 °C for 15 sec and 60 °C for 30 sec. The blocking effects of various WTBs were evaluated by analyzing the *C*_q_ values of WT-gDNA. The higher *C*_q_ values correlated with more optimal WTB blocking effects.

### Optimization of the internal competitive amplified fragments

To satisfy the thermodynamic driving force of *Taq* DNA polymerase, the amplification of an internal competitive amplified fragment (i.e., human *leptin* gene) was concomitantly performed in the WTB-PCR reaction mixture to further reduce or eliminate the nonspecific amplification of E19del WT-gene. Serial final concentrations of Lf-primer, Lr-primer and L-probe were additionally introduced into the optimized WTB-PCR reaction mixtures. Each reaction mixture contained 1x Platinum® Quantitative PCR SuperMix-UDG (Invitrogen), optimized concentrations of Ef-primer, Er-primer, E-probe and WTB oligonucleotides targeting E19del of the *EGFR* gene (Table 1), various final concentrations of Lf- and Lr-primer, and 150 nM L-probe (Table 1), and 100 ng of WT-gDNA. Reactions were performed on a CFX96 Real-Time PCR Detection System (Bio-Rad) under the following cycling conditions: incubation at 50 °C for 2 min, denaturation at 95 °C for 30 sec, and 50 or 60 cycles of 95 °C for 15 sec and 60 °C for 30 s.

### Comparative analysis of clinical samples

Sixty-two FFPE samples from NSCLC patients were analyzed by both WTB-PCR and commercially available ARMS-PCR (ACCB Biotech, Beijing, China). The ARMS-PCR was performed in accordance with the manufacturer’s instructions. Both reactions were performed on a CFX96 Real-Time PCR Detection System (Bio-Rad).

## Results

### Principle and potential applications of the developed WTB-PCR system

**Figure 2:**
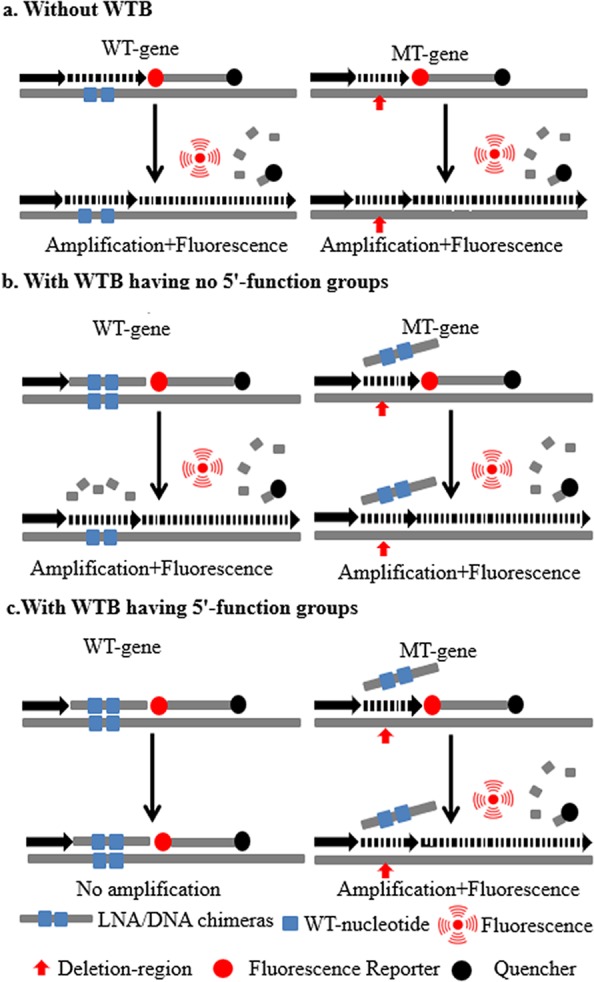
WTB-PCR assay using WTBs with and without 5′-functional groups. In the reaction mixture that lacked WTBs, the real-time traditional PCR could not be used to distinguish WT- and MT-alleles because both forms release fluorescence upon amplification (panel a). In the WTB-PCR system using WTBs that were positioned between forward and reverse primers, if the WTBs had no 5′-functional groups to resist the 5′ to 3′ exonuclease activity of *Taq* DNA polymerase (panel b), the WT- and MT-alleles could not be distinguished using traditional real-time PCR without WTBs (panel a). However, if the WTBs in the latter scenario harbored 5′-functional groups that could be used to resist the 5′ to 3′ exonuclease activity of *Taq* DNA polymerase (panel c), the amplification of WT-alleles could be effectively blocked thereby permitting the selective amplification of MT-alleles.

In the WTB-PCR system that was developed as part of this study, the WTBs were designed to locate between the forward and reverse primers. The potential advantage of this approach was that the MST probes labeled with various fluorescent reporters could be used to precisely distinguish certain mutations located in the complementary region of WTB oligonucleotides. Otherwise, LST probes that did not overlap with the WTB oligonucleotides could be used to simultaneously assay for the amplification of various MT-gene located in the complementary region of WTB oligonucleotides. However, to facilitate the release of fluorescence from the MST or LST oligonucleotides, the WTB oligonucleotides must be resistant to the 5′ to 3′ exonuclease activity of *Taq* DNA polymerase (Fig. 2). Compared with expensive PNA used for traditional WTB-PCR, LNA/DNA chimeras proved to be more cost effective and clinically acceptable^25^. However, traditionally employed LNA/DNA chimeras are not resistant to the 5′ to 3′ exonuclease activity of *Taq* DNA polymerase, and thus cannot be used as WTB oligonucleotides in WTB-PCR systems that selectively amplify MT-gene (Fig. 2b). Therefore, to enable LNA/DNA chimeras to be used as WTBs, two novel types of WTB oligonucleotide were developed as part of this study. The first type of WTB that was analyzed as part of this study contained one or more phosphorothioate bases at the 5′-end of the WTB oligonucleotides (WTB-2 to -4 in Table 1). The second WTB strategy utilized an inverted dT at the 5′-terminal base of the WTBs (WTB-5 in Table 1). All of the WTBs in this study including the traditional WTBs (WTB-1 in Table 1) were modified to include a C3-spacer or MGB that inhibited primer extension in the WTB-PCR system. In the PCR system without WTBs, both WT- and MT-gene could be amplified without any selectivity (Fig. 2a). For traditional WTBs (e.g., WTB-1 in Table 1), the WTB-PCR system did not facilitate discrimination of the WT- and MT-gene because the WTBs were hydrolyzed by the 5′ to 3′ exonuclease activity of *Taq* DNA polymerase following complementary hybridization with WT-gene (Fig. 2b). However, for WTBs with functional groups (i.e., phosphorothioate modifications or inverted dT), the WTB-PCR system enabled differentiation between the WT- and MT-gene because the WTB, which complementarily hybridized with WT-gene, could not be hydrolyzed by the 5′ to 3′ exonuclease activity of *Taq* DNA polymerase; this effectively blocked or eliminated the amplification of WT-alleles (Fig. 2c).

### Optimization of WTB-PCR

**Figure 3:**
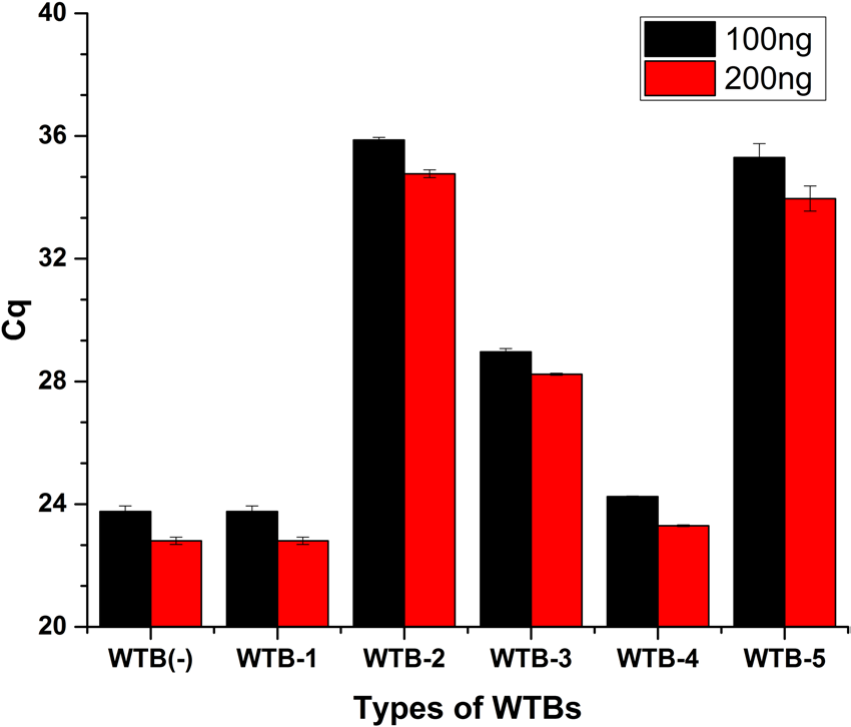
Blocking capacity of WTBs on the amplification of *EGFR* WT-alleles. The blocking capacity of WTB-1 to -5 targeting E19del of *EGFR* genes in reaction mixtures containing 100- and 200 ng of WT-gDNA. The blocking capacity of the different WTBs was evaluated using the *C*q values of WT-gDNA after amplification. The standard deviation(SD) values are indicated as error bars. WTB^−^ represents the negative control (i.e. without any WTBs).

To explore the blocking effects of WTBs, the concentrations of WT-gDNA isolates from healthy volunteers were precisely determined using the *leptin* qPCR system (Supplementary Fig. 1). In the reaction mixtures containing the various WTBs, higher *C*_q_ values equate to better blocking effects of WTBs. In preliminary experiments, specific quantities (100 and 200 ng) of WT-gDNA from EGFR E19del were co-amplified with certain concentrations (i.e., 500 nM) of various WTBs (Table 1) or without any WTBs (Fig. 3). Only the reaction mixture containing WTB-1 showed similar *C*_q_ values to the reaction mixture lacking any WTBs (WTB^−^ in Fig. 3); this result indicated that the WTBs that were not modified at the 5′ terminus were not resistant to the 5′ to 3′ exonuclease activity of *Taq* DNA polymerase, and therefore did not exhibit any blocking effects on the WT-gene of *EGFR* genes (Fig. 2b). However, all of the other WTB types (WTB-2 to -5 in Table 1) exhibited blocking effects because their *C*_q_ values were elevated compared with those of reaction mixtures without WTBs or with WTB-1 (Fig. 3). Because higher *C*_q_ values correlate with better WTB blocking effects, WTB-2 showed the strongest blocking effects as demonstrated by the mostly elevated *C*_q_ values (Fig. 3). The other 3 WTB types blocked the amplification of EGFR E19del WT-gene in the following order: WTB-5 > WTB-3 > WTB-4 (Fig. 3).

**Figure 4:**
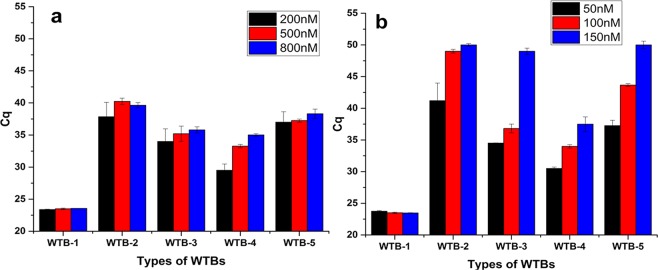
Optimization of the various oligonucleotides used in the reaction mixtures for the WTB-PCR systems. Screening various WTBs and optimization of the reaction system using 50 thermal cycles. Panel (a) comparison of the blocking effects of 5 WTBs at specified concentrations (200 nM, 500 nM and 800 nM). Panel (b) except for WTB-1, the blocking effects of various WTBs (WTB-2 to -5) were enhanced following the addition of internal competitive *leptin* amplification fragments. The *C*q value of 50 suggests that the amplification of WT-gDNA was completely blocked by WTBs.

To further explore the blocking effects, serial concentrations (200, 500, and 800 nM) of various WTBs were used in the reaction mixture containing 100 ng of *EGFR* WT-gDNA; these reactions required up to 50 thermal cycles (Fig. 4a). The results revealed no significant differences between the *C*_q_ values of reaction mixtures containing different concentrations of WTB-1. However, all of the other WTBs (WTB-2 to -5) exhibited blocking effects (after up to 50 thermal cycles) that correlated with the final concentrations of the WTBs, i.e. higher concentrations resulted in greater blocking capacities (i.e., higher *C*_q_ values), except for WTB-2 which had the greatest blocking capacity at a final concentrations of 500 nM (Fig. 4a).

As indicated in Fig. 4a, although WTB-2 to -5 showed blocking effects in relation to *EGFR* WT-gene, false-positive amplifications did occur in all reaction mixtures because all assays produced *C*_q_ values up to about 40 cycles. As described in our previous publications, these false-positive amplifications might be associated with the reduced fidelity of the *Taq* DNA polymerase used in the current reaction system^7,17,28^. Our previous publications further revealed that the afore-mentioned false-positive amplifications might be associated with the thermodynamic driving force of *Taq* DNA polymerase^7,17,28^. In order to overcome the afore-mentioned limitations, internal competitive amplification fragments (i.e., *leptin* genes; Table 1) were co-amplified with targeted *EGFR* genes^5,17,28,31^. Serial concentrations of primers and probes targeting human *leptin* genes that utilized in the *leptin* qPCR system (Supplementary Fig. 1). Except for reaction mixtures containing WTB-1 which exhibited no blocking effects, the *C*_q_ values of reaction mixtures containing additional *leptin* amplification fragments (Fig. 4b) were higher than those of reaction mixtures containing only oligonucleotides targeting *EGFR* genes (Fig. 4a). This result indicated that the afore-mentioned co-amplification approach enhanced the amplification specificities of the WTB-PCR approach^7,17,28^. However, compared with other analyzed reaction conditions, only the reaction mixture containing WTB-2 and 150 nM of Lf- and Lr-primer showed the capacity to fully eliminate the non-specific amplification of *EGFR* WT-gene in up to 50 thermal cycles.

**Figure 5:**
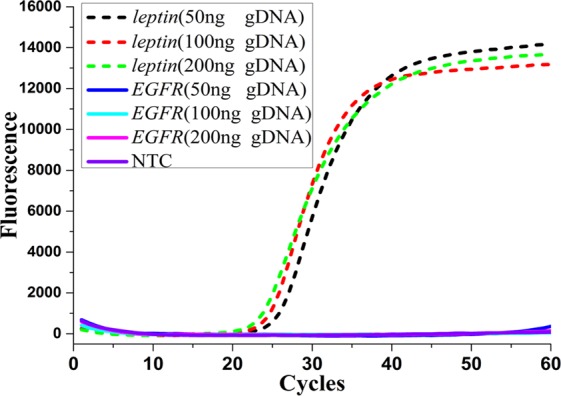
Confirmation of the optimum reaction conditions for WTB-PCR systems. Increasing amounts of WT-gDNA (50, 100, and 200 ng as indicated) were added into the WTB-PCR reaction mixtures containing oligonucleotides targeting both *EGFR* E19del and *leptin* genes at optimized reactions conditions. The number of thermal cycles was increased to 60 in these confirmatory assays. The short-dash and solid lines represent the amplification curves of internal competitive *leptin* fragments and targeted MT-alleles of E19del, respectively.

To further confirm the most optimal reaction conditions (Fig. 5), various quantities of WT-gDNA (50, 100, and 200 ng) were amplified by WTB-PCR. Moreover, the number of thermal cycles was increased to 60. The results revealed that amplification of up to 200 ng of WT-gDNA could be completely blocked for up to 60 thermal cycles (Fig. 5). After optimization, the following reaction conditions and reaction mixture compositions were used: 900 nM Ef- and Er-primer 400 nM E-probe, and 500 nM WTB-2 oligonucleotides targeting *EGFR* genes; 150 nM Lf- and Lr-primer, and 150 nM L-probe; 60 thermal reaction cycles.

### Sensitivity and selectivity of the optimized WTB-PCR reaction system

Sequencing was performed to confirm the WT- (SW-136 in Supplementary Fig. 2) and MT-QC (SW-137 to -147 in Supplementary Fig. 2) plasmid sequences. The SW-137 to -147 MT-QC plasmids represented the E746_A750del, E746_T751 > A, E746_S752 > V, E746_A750del, L747_A750 > P, L747_E749del, L747_S752del, L747_A750 > P, L747_P753 > S, L747_T751del and L747_T751 > P in-frame deletions most commonly observed in E19del mutants (Fig. 1 and Supplementary Fig. 2). All of the WT- and MT-QC plasmids were used in subsequent experiments to evaluate the optimum conditions for the WTB-PCR.

**Figure 6:**
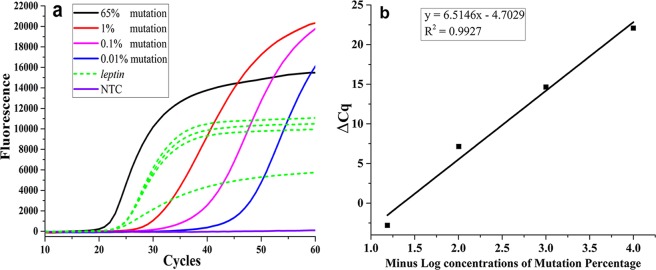
Properties of the current WTB-PCR system evaluated using various MT-QC plasmids. Panel (a) serial concentrations (3, 30, 300, 56,000 copies) of No. SW-143 MT-QC plasmids (i.e., E746_A750del) were spiked into a reaction mixture containing 100 ng of WT-gDNA; the latter concentrations represented selectivity values of 0.01%, 0.1%, 1% and 65%, respectively. The green dash lines represent the amplification curves of internal competitive amplified *leptin* genes. The other 10 MT-QC plasmids showed similar results as indicated in Supplementary Fig. 3. Panel (b) a standard curve was generated by plotting the average Δ*C*q values between *leptin* genes and SW-143 MT-QC plasmids against minus log percentage of MT-alleles (i.e., 0.01%, 0.1%, 1%, and 65%) generated in panel (a). The standard curves can be used to calculate the percentage of *EGFR* MT-alleles in clinical samples.

Serial dilutions of various MT-QC plasmids (3, 30, 300, 56,000 copies) were spiked into reaction mixtures containing 100 ng of WT-gDNA; the different concentrations of plasmids represented MT-gene selectivity percentages of 65, 1, 0.1 and 0.01%, respectively. The results showed that the selectivity of the current WTB-PCR sensitivity was down to 0.01%. Furthermore, the assay was capable of detecting as few as 3 MT-gene copies in a background of EGFR WT-gene copies (Fig. 6a and Supplementary Fig. 3). Moreover, based on the relationships between the *C*_q_ values for MT-gene and internal competitive amplified *leptin* genes, the Δ*C*_q_ values between *leptin* genes and MT-gene were used to calculate the percentage of MT-gene present in each reaction mixture (Fig. 6b).

Using gDNA from HCC827 cell lines harboring the E746-A750del mutations of E19del, confirmation experiments were performed to evaluate the optimal conditions for the WTB-PCR assay during the assessment of the 11 MT-QC plasmid types (Fig. 6, Supplementary Figs 3 and 4). Following serial dilution of MT- and WT-gDNA, we confirmed that the current WTB-PCR facilitated the detection of MT-gene with a selectivity value of 0.01% (Supplementary Fig. 4a). Using MT-gDNA from HCC827 cell lines as templates, the results revealed that the current WTB-PCR was sensitive enough to detect single copies of MT-gene (Supplementary Fig. 4b).

### Clinical applications of the WTB-PCR assay in assessment of FFPE samples

Sixty-two FFPE samples from NSCLC patients were analyzed using both the current WTB-PCR and commercially available ARMS-PCR assay. WTB-PCR detected E19del mutations in 32% (20/62) of samples. In contrast, ARMS-PCR only detected E19del mutations is 23% (14/62) samples. The consistency of both methods was 90% (56/62). Moreover, all of the E19del mutants detected by ARM-PCR were detected using WTB-PCR, while 6 mutants detected by WTB-PCR were not detected by ARMS-PCR. Based on the standard curves that were used to calculate the percentages of MT-gene present (Fig. 6b), the 6 additional mutant samples exhibited MT-gene percentages ranging from 0.02% to 0.41%; these results revealed that the WTB-PCR exceeded the detection limitations of traditional ARMS-PCR. Thus, the results of the WTB-PCR assay revealed that 28% (10 of 36) and 38% (10 of 26) of the analyzed samples from male and female NSCLC patients contained positive mutations, respectively (Supplementary Table 1).

## Discussion

The *EGFR* signal pathway is an important target for the treatment of NSCLC by TKI. Detection of drug-sensitive and -resistant mutations in exons 18 to 21 of *EGFR* genes is required for EGFR-TKI treatment of advanced NSCLC^32^. The *EGFR* mutations are predominantly positioned in exons 18–21, where the two most prevalent types of mutations are in-frame deletions in exon 19 (i.e., E19del) and an L858R point mutation in exon 21; the latter mutations account for less than 85% of all mutations^1,33,34^. Because of the heterogeneous nature of intra-tumor development, it was necessary to developed highly selective and sensitive methods to detect the low-abundance *EGFR* mutations present in various clinically available samples, such as FFPE tissue sections. WTB-PCR represents one of the most attractive assays among various available methods with the capacity to detect low-abundance mutations.

Traditional WTB-PCR systems can be divided into two groups in accordance with the complementary binding positions of WTB oligonucleotides against WT-gene. The first group involves the competitive binding of WTBs and forward primers (or, reverse primers) with the complementary templates^25^; these binding reactions result in overlapping sequences between the WTB and primer oligonucleotides. The second group uses WTBs that are positioned between the forward and reverse primers^7,17,28^; the WTB-PCR system used in this study (presented in Fig. 2c) belongs to the second group. In the first group, except for usage of allele-specific primers that was designed to overlap with WTB oligonucleotides, sequencing of the amplified fragments must be performed to precisely know the exact mutations^25^. Therefore, this group of WTB-PCR always had limited clinical applications. For the second group, it was possible to precisely know the exact mutations using MST oligonucleotides^17,26,28^. However, it must be stated that with the latter group the MST oligonucleotides (e.g., PNA) must be resistant the 5′ to 3′ exonuclease activity of *Taq* DNA polymerase. Otherwise, DNA polymerases (e.g., *Pfu* or DNA polymerase) that do not exhibit 5′ to 3′ exonuclease activity must be used in WTB-PCR system^25,26^. If DNA polymerases lacking 5′ to 3′ exonuclease activity are used in the second WTB-PCR group, the amplified fragments must eventually be sequenced in order to ascertain the type of mutation present in the targeted genes. In addition, the WTBs were always PNA oligonucleotides that could not be hydrolyzed by the 5′ to 3′ exonuclease activity of *Taq* DNA polymerase. However, the prohibitive cost of PNA oligonucleotides limits the clinical applications of such WTB-PCR assays. In our new publication, WTB oligonucleotides modified with phosphorothioate at the 5′-termini have been designed to specially detect missense mutations of KRAS gene using WTB-PCR assay^35^. In the present study, to explore more cost-effective WTB oligonucleotides that could be used in the latter WTB-PCR system for the detection of deletion mutations in targeted genes, one or more phosphorothioate bases and inverted dT at the 5′-end of the WTB oligonucleotides were designed. Studies have shown that phosphorothioate modification or the introduction of an inverted dT confers resistance against the 5′ to 3′ exonuclease activity of *Taq* DNA polymerase^36–39^.

In the present study, various WTB oligonucleotides were generated with phosphorothioate modifications or inverted dT at the 5′-terminal bases (WTB-2 to -5 in Table 1); an unaltered WTB (WTB-1 in Table 1) was used as a control. Only 4 WTBs harboring functional groups at the 5′-terminal bases (WTB-2 to -5 in Table 1) exhibited resistance to the 5′ to 3′ exonuclease activity of *Taq* DNA polymerase (Figs 4–6). However, the blocking effects were dependent on the properties of the functional groups in the afore-mentioned WTBs. WTB-2 exhibited the greatest blocking capacity, followed by WTB-5, -3, and -4. WTB-2 and -5 differed in relation to the functional group that they possessed; WTB-2 contained phosphorothioate as the 5′-terminal base while WTB-5 possessed an inverted dT at the same position. The blocking capacity of WTB-3 was greater than that of WTB-4. Notably, WTB-3 contains one additional LNA base suggesting that LNA bases positively influence the blocking capacity. The reason for the latter phenomenon might be due to the fact that LNA oligonucleotides confer significantly increased affinity to complementary DNA targets^40–42^. Moreover, following a comparison of the blocking capacities of WTB-2 and WTB-4, it is clear that the blocking effect is not necessarily proportional to the number of phosphorothioate bases. The WTB-2 and -3 oligonucleotides had one and three phosphorothioate bases, respectively; however, the blocking capacity of WTB-2 was greater than that of WTB-3. This suggested that the number of phosphorothioate bases might alter the associated structure thereby influencing binding efficiency.

Although WTBs could suppress WT-gene amplification, the false-positive amplification of WT-gene may also have been caused later on in the PCR due to the thermodynamic driving force of the DNA polymerase (Figs 3 and 4). Several studies have revealed that internal competitive amplified fragments can improve the specificity of target-gene amplification by satisfying the requirement for the thermodynamic driving force of DNA polymerase^7,17,28^. Therefore, the *leptin* gene which serves as an internal competitive amplified fragment was introduced in the current WTB-PCR system. One of the primary benefits associated with the inclusion of the internal competitive *leptin* amplification fragment was the increased specificity observed in the WTB-PCR system (even when less than 60 thermal cycles were used). When WT-gene spiked with increasing concentrations of MT-gene were assessed, the results revealed that the WTB-PCR system facilitated the detection of MT-gene at percentages as low as 0.01% with a sensitivity limit of up to a single copy of the MT-gene. Moreover, the WTB-PCR system inhibited the amplification of WT-gDNA when up to 200 ng of the latter DNA was present; this capacity is extremely useful when analyzing DNA isolates from various tissue samples such as FFPE sections.

Previous publications have indicated that methods exhibiting increased selectivity facilitate the detection of a greater number of positive mutations in samples. For example, the percentage of positive mutations detected by PNA clamping (i.e., 18.84%) was higher than that of direct DNA sequencing (i.e., 7.24%)^43^. Furthermore, the scorpion-ARMS identified a greater number of samples (i.e., 25%) harboring *EGFR* mutations compared with traditional DNA sequencing approaches^11^. Compared with ARMS-PCR which exhibits reduced selectivity (i.e., up to 1%), the current WTB-PCR exhibits greater selectivity (0.01%) and also enabled the detection of a greater number of positive E19del mutations in samples. It was observed that the positive percentage of WTB-PCR was 32% (20 of 62), while that of ARMS-PCR was only about 23% (14 of 62). The MT-gene percentages of the additional six mutation positive samples being detected only by WTB-PCR were ranged at 0.02–0.41%, which had exceeded the detection limitations of ARMS-PCR. Moreover, analysis of results generated by the WTB-PCR system revealed that the *EGFR* E19del mutation of female was higher than that of the male in NSCLC which was consistent with previous studies^34,44,45^.

In conclusion, the WTB-PCR system may prove to be a robust tool for genotyping clinical samples. In our laboratory, similar WTB-PCR assays targeting other oncogenic mutations that play important roles in personalized treatments such as *KRAS* and *BRAF* genes are being developed.

## Supplementary information

supplementary material
