# Supplementary material for: Sensitive detection of low-abundance in-frame deletions in EGFR exon 19 using novel wild-type blockers in real-time PCR
Source: Sci Rep. 2019 Jun 4;9:8276. doi: 10.1038/s41598-019-44792-1 (PMC6547704; doi:10.1038/s41598-019-44792-1)
Supplement: Supplementary file 1 — supplementary material [file 41598_2019_44792_MOESM1_ESM.doc]

**SUPPLEMENTARY INFORMATION**

Sensitive detection of low-abundance in-frame deletions in EGFR exon 19 using novel wild-type blockers in real-time PCR

Xiao-Dong Ren, Ding-Yuan Liu, Hai-Qin Guo, Liu Wang, Na Zhao, Ning Su, Kun Wei, Sai Ren, Xue-Mei Qu, Xiao-Tian Dai, Qing Huang

**Table 1.** The information of FFPE samples and the detection results

| **ID** | **Sex** | **Age** | **DNA concentrations (ng/ul)** | **OD260/280** | **Results of ARMS-PCR** | **WTB-PCR** | | | | |
| --- | --- | --- | --- | --- | --- | --- | --- | --- | --- | --- |
|  |  |  |  |  |  | Cq values of target gene | Cq values of leptin gene | Results | ΔCq | Mutations abundance |
| **1** | Female | 52 | 109.84 | 1.92 | N | 0 | 27.87±0.141 | N | - | 0 |
| **2** | Female | 61 | 32.23 | 1.94 | N | 0 | 27.61±0.358 | N | - | 0 |
| **3** | male | 62 | 53.3 | 1.95 | N | 0 | 28.22±0.605 | N | - | 0 |
| **4** | male | 58 | 21.76 | 1.93 | N | 0 | 28.05±0.412 | N | - | 0 |
| **5** | male | 61 | 44.33 | 1.78 | N | 0 | 29.02±0.273 | N | - | 0 |
| **6** | male | 76 | 96.77 | 1.78 | N | 0 | 27.09±0.141 | N | - | 0 |
| **7** | male | 50 | 65.53 | 1.84 | N | 43.26±0.4 | 29.16±0.041 | P | 14.1 | 0.13% |
| **8** | male | 44 | 40.1 | 1.83 | N | 0 | 28.45±0.094 | N | - | 0 |
| **9** | male | 61 | 13.4 | 1.84 | P | 33.49±0.393 | 30.14±0.014 | P | 3.35 | 5.81% |
| **10** | male | 66 | 53.63 | 1.84 | P | 29.97±1.207 | 28.39±0.082 | P | 1.58 | 10.85% |
| **11** | male | 57 | 85.31 | 1.9 | N | 0 | 27.8±0.042 | N | - | 0 |
| **12** | male | 49 | 17.49 | 1.93 | N | 0 | 31.12±0.041 | N | - | 0 |
| **13** | male | 65 | 41.59 | 1.93 | N | 0 | 28.75±0.094 | N | - | 0 |
| **14** | Female | 34 | 14.35 | 2.07 | P | 32.72±0.4 | 29.89±0.014 | P | 2.83 | 6.98% |
| **15** | male | 59 | 38.87 | 1.94 | N | 0 | 28.93±0.082 | N | - | 0 |
| **16** | male | 40 | 31.31 | 1.76 | N | 0 | 29.86±0.042 | N | - | 0 |
| **17** | male | 58 | 64.1 | 1.88 | N | 0 | 27.85±0.202 | N | - | 0 |
| **18** | male | 67 | 25.35 | 1.86 | N | 44.65±0.471 | 29.14±0.070 | P | 15.51 | 0.08% |
| **19** | male | 64 | 7.48 | 1.86 | P | 27.18±0.165 | 30.53±009 | P | -3.35 | 61.99% |
| **20** | male | 67 | 15.28 | 1.93 | P | 37.06±0.393 | 30.61±0.071 | P | 6.45 | 1.95% |
| **21** | Female | 61 | 33.56 | 1.84 | P | 36±0.207 | 27.87±0.042 | P | 8.13 | 1.10% |
| **22** | Female | 50 | 14.34 | 1.78 | N | 0 | 28.87±0.069 | N | - | 0 |
| **23** | Female | 52 | 34.43 | 1.87 | N | 0 | 29.45±0.074 | N | - | 0 |
| **24** | Female | 50 | 38.95 | 1.93 | N | 0 | 27.91±0.069 | N | - | 0 |
| **25** | Female | 72 | 27.34 | 2 | N | 0 | 30.44±0.665 | N | - | 0 |
| **26** | Female | 37 | 14.65 | 1.98 | P | 29.25±0.4 | 31.07±0.098 | P | -1.82 | 36.10% |
| **27** | Female | 46 | 25.63 | 2.01 | N | 0 | 28.12±0.043 | N | - | 0 |
| **28** | Female | 49 | 80.56 | 1.84 | N | 0 | 26.73±0.063 | N | - | 0 |
| **29** | Female | 56 | 20.95 | 1.9 | N | 0 | 30.54±0.235 | N | - | 0 |
| **30** | Female | 56 | 36.72 | 1.89 | N | 41.07±0.471 | 30.25±0.053 | P | 10.82 | 0.41% |
| **31** | Female | 47 | 36.94 | 1.98 | N | 0 | 29±0.069 | N | - | 0 |
| **32** | Female | 63 | 58.12 | 1.88 | N | 45.96±0.165 | 27.02±0.326 | P | 18.94 | 0.02% |
| **33** | male | 66 | 55.3 | 1.9 | N | 0 | 26.98±0.254 | N | - | 0 |
| **34** | male | 57 | 79.16 | 1.92 | N | 0 | 26.55±0.070 | N | - | 0 |
| **35** | Female | 60 | 54.67 | 1.96 | N | 42.5±0.393 | 26.83±0.026 | P | 15.67 | 0.07% |
| **36** | Female | 49 | 30.37 | 1.77 | N | 0 | 30.88±0.056 | N | - | 0 |
| **37** | Female | 61 | 197.5 | 1.94 | N | 0 | 28.53±0.604 | N | - | 0 |
| **38** | Female | 58 | 28.45 | 1.89 | P | 35.62±0.207 | 20.82±0.026 | P | 14.8 | 0.10% |
| **39** | Female | 47 | 39.31 | 2.11 | N | 0 | 29.74±0.443 | N | - | 0 |
| **40** | Female | 62 | 32.5 | 2.05 | N | 0 | 28.39±0.079 | N | - | 0 |
| **41** | Female | 50 | 19.46 | 2.17 | N | 0 | 29.29±0.438 | N | - | 0 |
| **42** | Female | 63 | 19.67 | 1.78 | N | 0 | 31.14±0.365 | N | - | 0 |
| **43** | Female | 43 | 56.22 | 1.81 | P | 27.92±0.273 | 28.14±0.665 | P | -0.22 | 20.50% |
| **44** | Female | 59 | 110.15 | 1.82 | N | 0 | 27.8±0.925 | N | - | 0 |
| **45** | male | 53 | 9.7 | 1.9 | N | 0 | 30.17±0.020 | N | - | 0 |
| **46** | Female | 69 | 70.46 | 1.91 | N | 0 | 26.93±0.035 | N | - | 0 |
| **47** | Female | 80 | 86.51 | 1.87 | N | 0 | 27.79±0.085 | N | - | 0 |
| **48** | Female | 51 | 155.24 | 1.84 | N | 0 | 25.84±0.016 | N | - | 0 |
| **49** | Female | 55 | 27.34 | 1.92 | P | 29.18±0.412 | 28.16±0.683 | P | 1.02 | 13.24% |
| **50** | Female | 52 | 88.75 | 1.76 | P | 30.15±0.562 | 27.61±0.244 | P | 2.54 | 7.80% |
| **51** | Female | 73 | 36.06 | 1.91 | N | 0 | 26.26±0.128 | N | - | 0 |
| **52** | Female | 41 | 26.95 | 1.73 | N | 0 | 28.97±0.123 | N | - | 0 |
| **53** | Female | 46 | 57.79 | 1.86 | P | 26.71±0.141 | 27.59±0.008 | P | -0.88 | 26.30% |
| **54** | Female | 72 | 74.18 | 1.88 | P | 28.15±0.412 | 27.18±0.022 | P | 0.97 | 13.49% |
| **55** | Female | 43 | 56.51 | 1.83 | N | 0 | 25.92±0.332 | N | - | 0 |
| **56** | Female | 64 | 20.24 | 1.83 | N | 0 | 29.27±0.190 | N | - | 0 |
| **57** | Female | 57 | 38.12 | 1.87 | N | 0 | 29.64±0.452 | N | - | 0 |
| **58** | Female | 39 | 38.04 | 1.8 | N | 0 | 28.92±0.152 | N | - | 0 |
| **59** | Female | 64 | 64.1 | 1.92 | P | 26.06±0.605 | 27.54±0.086 | P | -1.48 | 31.99% |
| **60** | male | 64 | 85.5 | 1.93 | N | 44.21±0.358 | 27.59±0.011 | P | 16.62 | 0.05% |
| **61** | Female | 53 | 25.95 | 1.93 | N | 0 | 29.9±0.472 | N | - | 0 |
| **62** | male | 58 | 15.39 | 1.8 | N | 0 | 29.45±0.028 | N | - | 0 |

A total of 62 FFPE samples from NSCLC patients were analyzed by both WTB-PCR and ARMS-PCR. N and P represent negative and positive results respectively.

# Figure 1 The standard curve for the *leptin* qPCR system.





The standard curve was generated by plotting the average Cq values of *leptin* against the log starting quantity (i.e., 100, 1,000, 5,000, 25,000, 50,000 pg) of WT-gDNA from healthy volunteers.

# Figure 2 Sequencing chromatograph of WT- and MT-QC plasmids for the *EGFR* E19del.





The sequencing chromatograph of the WT-alleles (SW-136) and 11 types of the hot E19del mutations (SW-137 to 147). The red arrows indicate the position of various in-frame deletions in exon 19 of *EGFR* genes (E19del).

**Figure 3** Properties of the current WTB-PCR system using QC plasmids as templates.





The sensitivity and selectivity of the other 10 MT-QC plasmids (SW-137 to -142, and SW-144 to -147) in reaction mixtures containing serial percentages of MT-alleles as indicated; these assays were performed as described in Figure 6. The green dash lines represent the internal competitive amplified *leptin* genes.

# Figure 4 Properties of the current WTB-PCR system using MT-gDNA as template.







Panel a: Reaction mixture containing serial percentages (100%, 10%, 1%, 0.1%, and 0.01% as indicated) of MT-alleles coming from MT-gDNA; these reactions were performed at optimized reaction conditions. The serial percentages of MT-alleles were prepared by mixing specified amounts of MT-gDNA and WT-gDNA, and the total amount of gDNA was 100 ng. The green dash lines represent the internal competitive amplified *leptin* genes. Panel b: Serial concentrations (50 ng, 5ng, 500 pg, 50 pg, 10 pg, and 5 pg as indicated) of MT-gDNA from HCC-827 cell lines were amplified at optimized reaction conditions. The green dash lines represent the internal competitive amplified *leptin* genes.
